# Transcription factors WT1 and p53 combined: a prognostic biomarker in ovarian cancer

**DOI:** 10.1038/s41416-018-0191-x

**Published:** 2018-07-30

**Authors:** Julia H. Carter, James A. Deddens, Gretchen Mueller, Thomas G. Lewis, Mariah K. Dooley, Michelle C. Robillard, Molly Frydl, Lydia Duvall, Jackson O. Pemberton, Larry E. Douglass

**Affiliations:** 1 Wood Hudson Cancer Research Laboratory, Newport, KY 41071 USA; 2 Department of Mathematical Sciences, University of Cincinnati, Cincinnati, OH 45202 USA; 3 St. Elizabeth Healthcare, Edgewood, KY 41018 USA

**Keywords:** Ovarian cancer, Immunohistochemistry

## Abstract

**Background:**

New approaches to ovarian cancer are needed to improve survival. Wilms’ tumour 1 (WT1) is a tumour-associated antigen expressed in many ovarian cancers. P53 is also often altered. The clinical significance of the combined expression of these two transcription factors has not been studied.

**Methods:**

One hundred ninety-six ovarian tumours were classified histopathologically. Tumours were stained for WT1 and p53 immunohistochemically. Stains were analysed according to tumour type, grade and FIGO stage. Kaplan–Meier analyses on 96 invasive carcinomas determined whether categorical variables were related to survival.

**Results:**

WT1 and p53 were related to ovarian tumour type, grade, FIGO stage and patient survival. Uniform nuclear p53 expression was associated with invasion and WT1 expression was associated with advanced grade, FIGO stage and poor survival. When WT1 and p53 were both in the age-adjusted Cox model, WT1 was significant while p53 was not. When we combined tumours expressing WT1 and p53, then adjusted for age and tumour subtype, the hazard ratio compared to tumours without WT1 and with normal p53 was 2.70; when adjusted for age and FIGO stage, the hazard ratio was 2.40.

**Conclusions:**

WT1, an antigen target, is a biomarker for poor prognosis, particularly when combined with altered p53.

## Introduction

Epithelial ovarian cancer (OvCa) is the leading cause of death from gynaecologic cancers and has the highest mortality rate of any female reproductive cancer. In 2012, worldwide incidence of ovarian cancer was 239,000 new cases, with 152,000 ovarian cancer deaths.^1^ Most patients are diagnosed at late stages and have a poor prognosis. Radical surgery and platinum-based chemotherapy are the primary treatment. When relapse occurs, there are few treatment options. The five-year survival rate of women with distant metastases is only 17%.^2^ These statistics highlight the need for new approaches to OvCa therapy.

Epithelial OvCa is a heterogeneous disease with multiple subtypes that arise either from extraovarian tissues such as the fallopian tube, endometrium, endometriosis, the gastrointestinal tract, or in some cases from the ovarian surface epithelium or inclusion cysts arising from this surface epithelium.^3,4^ The morphological subtypes of epithelial OvCa include serous, endometrioid, clear cell and mucinous. Histopathological, molecular and genetic studies suggest that these tumours can be divided into two broad categories: type 1, which are indolent, can develop from precursor lesions such as borderline tumours, and are usually confined to the ovary at diagnosis; and type 2, which can arise de novo from the tubal or ovarian surface epithelium, are genetically unstable, rapidly progress and are often diagnosed at late stages.^5^ Type 1 tumours include low-grade serous and endometrioid, mucinous and clear cell carcinomas. Type 2 tumours include high-grade serous and endometrioid carcinomas, undifferentiated carcinomas and carcinosarcomas. Degree of differentiation determines grade with borderline tumours deviating minimally from benign tissues, grade 1 tumours being well differentiated, grade 2 tumours being moderately differentiated and grade 3 tumours being poorly differentiated. Surgical staging of OvCa is by the International Federation of Gynaecology and Obstetrics (FIGO) classification: stage I tumour is localised to one or both ovaries; stage II tumour is associated with pelvic extension; stage III tumour has spread into the abdominal cavity or to retroperitoneal lymph nodes; stage IV tumour is present in liver parenchyma or distant metastases.^6^ Although tumour subtype, grade and stage are related to survival, none offer specific targets for therapy.

Immunotherapy is a treatment modality that could be efficacious in OvCa. OvCa is associated with an immune response in many patients.^7–11^ Therapeutic vaccines are the most studied immunotherapeutic strategy in epithelial OvCa.^11,12^ Any mutant, overexpressed or abnormally expressed protein in cancer cells can be a target for cancer vaccines and/or T cell therapy.^13^ Missense mutations in *TP53* are common in OvCa and, due to gain of function, can harbour a poor prognosis in some, but not all, studies.^3,14–16^ Similarly, the Wilms’ tumour protein (WT1) is expressed in many OvCas and is a poor prognostic factor.^17,18^ Both WT1 and p53 are tumour-associated antigens.^13^ WT1 is ranked first in pilot prioritisation out of 75 cancer antigens based on predefined criteria, including therapeutic efficacy and immunogenicity.^13^ Molecular studies in multiple models indicate that these two transcription factors, p53 and WT1, interact both physically and functionally.^19–23^ However, the clinical significance of p53 and WT1 expression combined has not been studied in OvCa and hence the potential for the combined expression to be a clinically useful prognostic factor and a predictive marker for response to immunotherapy is not known.

Here we report that the combination of these two immunohistochemically (IHC) detected transcription factors and tumour-associated antigens diffusely expressed in some OvCas, varies both within and between morphologic subtypes of ovarian tumours and that IHC detection of these tumour-associated antigens in OvCa could be useful biomarkers allowing better prognostication and patient selection for newer immunologic approaches targeting WT1 for therapy of lethal OvCa.

## Materials and methods

### Tissues studied

Archived, formalin fixed, paraffin-embedded (FFPE) tissues were donated to Wood Hudson Cancer Research Laboratory by St. Elizabeth Healthcare in Northern Kentucky and are preserved in the environmentally controlled Wood Hudson Biospecimen Repository. The study had the approval of the Institutional Review Board of St. Elizabeth Healthcare. Wood Hudson Cancer Research Laboratory has an HIPAA waiver from St. Elizabeth Healthcare. In addition to the tissues from St. Elizabeth Healthcare, FFPE tissues were obtained from the Cooperative Human Tissue Network, Birmingham, Alabama.

**Fig. 1:**
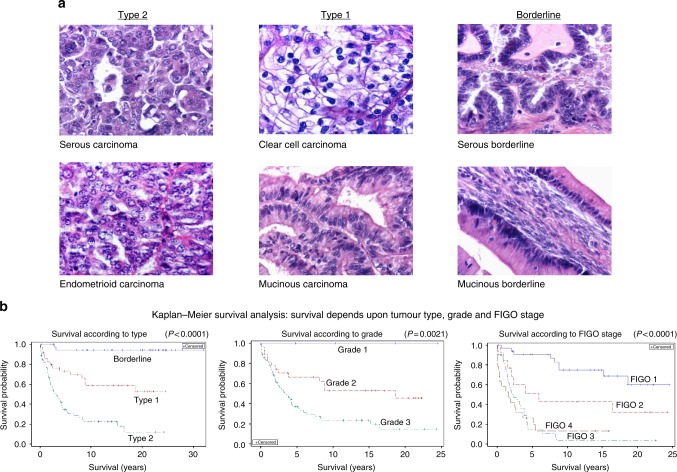
**a** Histopathology of ovarian tumour subtypes (×400): Serous carcinoma. Most frequent ovarian carcinoma. It can be either high grade (type 2) or low grade (type 1). Endometrioid carcinoma. Can be either high grade (type 2) with few glandular spaces, or low grade (type 1) with glandular spaces suggestive of endometrial glands. Clear cell carcinoma. This type 1 carcinoma contains cells with clear cytoplasm. Mucinous carcinoma. Mucin containing cells in this type 1 carcinoma are arranged in a disorderly fashion. Serous borderline. Tumour cells contain eosinophilic or amphophilic cytoplasm and are columnar or cuboidal in shape and line a cystic cavity in an orderly fashion. Mucinous borderline. Tumour cells are orderly in their arrangement, line a cystic cavity, and contain much mucin, which maybe either eosinophilic or basophilic. **b** Kaplan–Meier survival analysis demonstrates that patient survival depends upon ovarian tumour type, grade, and FIGO stage (*p* < 0.0001)

A total of 196 archived FFPE surgical specimens obtained from surgeries performed between 1981 and 2006 were stained and evaluated. Ovarian tumours were classified according to subtype by a Board Certified pathologist (LED). For purposes of analysis, ovarian tumours were grouped according to the classification system proposed by Kurman and Shih^4,5^ (Table 1A and Fig. 1a). Type 1 tumours (total, *N* = 51) were low-grade serous (*N* = 9), low-grade endometrioid (*N* = 6), low-grade mixed epithelial (*N* = 4), clear cell (*N* = 14) and mucinous carcinomas (*N* = 18). Type 2 tumours (*N* = 96) were high-grade serous (*N* = 64), high-grade endometrioid (*N* = 25), high-grade mixed epithelial (*N* = 4) and carcinosarcoma (*N* = 3). In addition, 49 borderline ovarian tumours were studied including serous borderline tumours (*N* = 28), mucinous borderline tumours (*N* = 20) and one mixed epithelial borderline tumour.

**Table 1:** Patient characteristics (subtype, FIGO stage, and tumour grade are not 100% correlated with death from OvCa)

A. All patients (*N* = 196)						
Tumour subtype	*N*	Average age (+/-SEM)	FIGO stage I, II *n* (%)	FIGO stage III, IV *n* (%)	Tumour grade 1, 2 *n* (%)	Tumour grade 3 *n* (%)
Type 2	96	64.0 ± 1.4	28 (29.2%)	68 (70.2%)	0	96 (100%)
Type 1	51	58.0 ± 1.9	34 (66.7%)	17 (33.3%)	45 (88.2%)	6 (11.8%)
Borderline	49	49.2 ± 2.4	40^a^ (81.6%)	9^a^ (18.4%)	n/a	n/a

B. Patients with clinical follow-up (*N* = 137)							
Tumour subtype	*N*	Average age (+/-SEM)	FIGO stage I, II *n* (%)	FIGO stage III, IV *n* (%)	Tumour grade 1, 2 *n* (%)	Tumour grade 3 *n* (%)	Died OvCa *n* (%)
Type 2	61	64.7 ± 1.7	18 (29.5%)	43 (70.5%)	0	96 (100%)	46 (75.4%)
Type 1	35	58.1 ± 2.5	29 (82.8%)	6 (17.1%)	32 (91.4%)	3 (8.6%)	13 (39.4%)
Borderline	41	50.0 ± 2.7	36^a^ (87.8%)	5^a^ (12.2%)	n/a	n/a	2 (5%)

Only patients with type 2 and type 1 ovarian cancers (*N* = 96) were used in survival analyses.

^a^Implants not metastases

Clinical follow-up was obtained with the assistance of Tumour Registrars at St. Elizabeth Healthcare from The Kentucky Cancer Registry (part of the SEER network) for 134 of the 136 patients with tissues donated by St. Elizabeth Healthcare; two patients were lost to follow-up. Most patients with type 2 and type 1 tumours received further treatment (87% and 65%, respectively) while patients with borderline tumours rarely received further treatment (7%). Follow-up information was available for up to 25 years (median 3.9 years). For type 1 patients (*N* = 35), follow-up time ranged from 0 to 25 with a median of 8.84, for type 2 patients (*N* = 61) the range was also 0–25 with a median of 2.67. Seventy-five percent of patients with type 2 OvCa in this study died from ovarian cancer while 40% of type 1 patients died from OvCa.

Clinical follow-up was not available for the 59 patients with tumours obtained from the Cooperative Human Tissue Network. Since only two patients with borderline ovarian tumours died from their disease, all survival analyses were on 96 patients with type 1 and type 2 OvCas with follow-up data.

### Immunohistochemistry

Air-dried 5 micron paraffin sections were dewaxed in Clearite, and rehydrated through a graded series of alcohols to distilled water. They were placed in tris-buffered saline (TBS) containing 0.05% Tween. Following rehydration, antigen retrieval was with 1× Dako Antigen Retrieval Solution, pH 9.0 (Dako, Carpinteria, CA, USA; cat. no.: S2367), heated to 97–98 °C in a water bath for 20 min. Sections were allowed to cool for 20 min, and then rinsed in deionised water and placed in TBS-Tween. Adjacent sections were stained immunohistochemically for WT1, p53 or IgG negative controls. WT1 was detected with a mouse monoclonal antibody clone 6F-H2 against the N terminus amino acids, 1−181 (Dako; cat. no.: M3561, 1.706 microg/mL) or mouse IgG1 control (Dako; cat. no.: X0931, 1.706 microg/mL). p53 expression was detected with a mouse monoclonal antibody, clone DO-7 (Dako; cat. no.: M7001, 0.137 microg/mL) or a mouse IgG2B control (Dako; cat. no.: X0944, 0.137 microg/mL). Others have reported that this antibody is suitable for detecting all p53 variants identified by massive parallel sequencing.^16^ Both antibodies and their IgG controls were diluted with Dako diluent (cat. no.: S0809). Sections were stained for 60 min using a Dako autostainer and the LSAB2 staining system (Dako) with diaminobenzidine as chromagen. Immunohistochemically stained sections were counterstained with methyl green, dehydrated in butanol and Clearite, and coverslipped with Permount (Fisher Healthcare, Hanover Park, IL, USA). A slide of fallopian tube from a single case was included in every IHC run as a staining control along with a buffer control.

Immunohistochemically stained sections were evaluated by two observers (L.E.D. and J.H.C.) using a Nikon Coolscope (Nikon Instruments, New York, NY, USA). Tumours were considered positive for WT1 if 75–100% of their nuclei were stained intensely (3+) by the WT1 antibody (Histoscore = Area × Intensity = 2.7). Tumours were considered to have altered p53 expression if either 75–100% of their nuclei were stained strongly (2.5–3 + intensity; Histoscore = 2.25–2.7) by the p53 antibody or if the tumour was totally negative.^16,24,25^ Tumours were considered to have normal p53 expression if there were variably stained scattered positive nuclei in the tumour (more than 10% but less than 75% of nuclei). Observers were blinded to patient outcome.

### Study design

This was a retrospective study in which patients with ovarian tumours were selected from tumour registries without knowledge of clinical outcome. Although surgical pathology reports were obtained for each case, a single pathologist (L.E.D.) reviewed, diagnosed and graded an H&E-stained slide of each specimen studied. Clinical information obtained as part of the study included patient age, date of diagnosis, stage of disease, date of recurrence, site of metastases, if patient had additional forms of cancer, date of death (or date known to be alive) and if death was due to OvCa. Variables analysed were subtype of OvCa, grade of tumour, stage of disease, nuclear WT1 expression, normal and altered p53 expression, and survival.

### Statistical analysis methods

A total of 196 archived FFPE surgical specimens of ovarian tumours were classified histopathologically. Tumours were stained for expression of WT1 and p53 immunohistochemically. Stain patterns were analysed according to tumour type, grade and FIGO stage. Kaplan–Meier analyses (log-rank tests) were conducted on 96 patients with type 1 and type 2 OvCa with follow-up data, to determine whether categorical variables were related to survival and to obtain estimates of survival curves. Cox proportional hazards regression models were conducted to determine hazard ratios of categorical variables as well as to determine interactions and to adjust for possible confounding variables (e.g., age).

## Results

### Altered p53 expression is related to ovarian tumour morphology

Table 2A gives the p53 immunohistochemical staining patterns of the 196 tumours in our data set according to morphologic cell type. The normal p53 stain pattern (variable stain in 10–75% nuclei) was found in nearly all clear cell OvCa and borderline serous tumours, and also in 31–67% of borderline mucinous tumours, and serous, mucinous and endometrioid carcinomas and carcinosarcoma subtypes (Table 2A). An absence of p53 immunoreactivity was found in 50% and 22%, respectively, of borderline mucinous tumours and mucinous OvCa. Only 10–12% of borderline serous tumours and invasive serous carcinomas and 29% of endometriod OvCa were without p53 immunoreactivity. In contrast, 22–67% of serous, mixed epithelial, endometriod and carcinosarcomas had near uniform nuclear immunostaining for p53. Notably, near uniform nuclear immunostaining for p53 was found only in invasive OvCa and was not found in borderline serous tumours.

**Table 2:** Biomarker expression according to morphological subtype

A. p53 status according to morphological subtype				
Subtype histoscore (area × intensity)^a^	*N*	Abnormal loss (*N*) (%) p53 = 0	Normal p53 (*N*) (%) p53>0.1<2.25	Abnormal uniform stain (*N*) (%) p53 = 2.25–2.7
Borderline serous	29	3 (10.3%)	26 (89.7%)	0
Type 1 serous	9	2 (22.2%)	5 (55.6%)	2 (22.2%)
Type 2 serous	64	7 (10.90%)	19 (29.7%)	38 (59.4%)
Borderline mucinous	18	10 (50%)	9 (45%)	1 (5%)
Type 1 mucinous	14	4 (22.2%)	12 (66.7%)	2 (11.1%)
Borderline mixed epithelial	1	0	1 (100%)	0
Type 1 mixed epithelial	4	0	1 (25%)	3 (50%)
Type 2 mixed epithelial	4	0	0	4 (100%)
Type 1 endometrioid	6	3 (50%)	3 (50%)	0
Type 2 endometrioid	25	6 (24%)	10 (40%)	9 (36%)
Type 2 carcinosarcoma	3	0	1 (33.3%)	2 (66.7%)
Type 1 clear cell	14	0	13 (92.9%)	1 (7.1%)

B. WT1 status according to morphological subtype			
Subtype	*N*	WT1− (*N*) (%)	WT1+ (*N*) (%)
Borderline serous	28	2 (7.1%)	26 (92.9%)
Type 1 serous	9	7 (77.8%)	2 (22.2%)
Type 2 serous	64	5 (7.8%)	59 (92.8%)
Borderline mucinous	20	20 (100%)	0
Type 1 mucinous	18	16 (88.9%)	2 (11.1%)
Borderline mixed epithelial	1	1 (100%)	0
Type 1 mixed epithelial	4	2 (50%)	2 (50%)
Type 2 mixed epithelial	4	0	4 (100%)
Type 1 endometrioid	6	5 (83.3%)	1 (16.7%)
Type 2 endometrioid	25	9 (36%)	16 (64%)
Type 2 carcinosarcoma	3	1 (33.3%)	2 (66.7%)
Type 1 clear cell	14	13 (92.9%)	1 (7.1%)

^a^See Materials and Methods

### Nuclear expression of WT1 is related to ovarian tumour morphology

WT1 expression according to ovarian tumour morphology is given in Table 2B. The known association of nuclear WT1 expression with serous differentiation in the ovary was found in both borderline serous ovarian tumours and type 1 and type 2 serous OvCa. Almost all of 64 high-grade serous carcinomas expressed WT1 in 75–100% of their nuclei. OvCa with other morphologic differentiation also expressed WT1. Approximately half of ovarian endometrioid carcinomas expressed WT1 as well as most mixed epithelial cell OvCa and most carcinosarcomas. Two mucinous and one clear cell OvCa also expressed nuclear WT1. Although serous borderline tumours did not express uniform p53 stain, all serous borderline tumours, which are non-invasive and are minimally deviated histologically from normal ovarian tissues, expressed nuclear WT1.

### Altered p53 and nuclear WT1 expression combined are related to tumour type, grade and stage

**Fig. 2:**
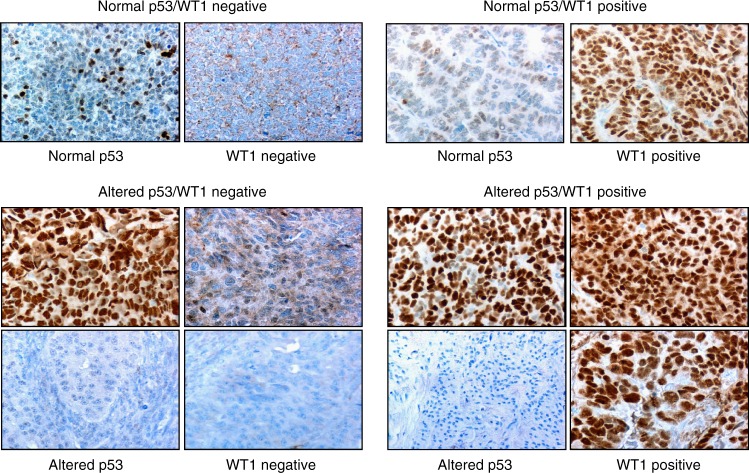
Combinations of WT1 and p53 expression in invasive OvCa (×400). Expression of p53 was considered “Normal” when nuclei were variably stained and scattered positive nuclei were seen in more than 10% but less than 75% of nuclei (nuclear histoscore area × intensity p53 > 0.1 < 2.25). p53 expression was considered “Altered” when tumour cells were strongly positive 2^+^–3^+^ in 75–100% nuclei (abnormal uniform stain, upper image, nuclear histoscore area × intensity p53 = 2.25–2.7) or completely devoid of stain (abnormal loss lower image, nuclear histoscore area × intensity p53 = 0). WT1 was considered positive when 75–100% of nuclei expressed this transcription factor. Images illustrate the four combinations of expression of these two transcription factors and tumour-associated antigens

Four combinations of p53 IHC stain pattern and nuclear WT1 expression were found in OvCa: normal p53 stain pattern and no nuclear WT1 expression (p53/WT1^−^); altered p53 stain pattern and no nuclear WT1 expression (p53^alt^/WT1−); normal p53 stain pattern and nuclear WT1 expression (p53/WT1^+^); and altered p53 IHC stain pattern and nuclear WT1 expression (p53^alt^/WT1^+^) (Fig. 2). Taken together, these four combinations of IHC detected p53 and WT1 nuclear expression were related to ovarian tumour type, ovarian tumour stage and ovarian tumour grade (Table 3A, B, C).

**Table 3:** WT1 and altered p53 in OvCa

A. Altered p53 and WT1 expression differ significantly in OvCa according to subtype (*n* = 196) (*p* < 0.0001)					
Tumour subtype	*n*	p53/WT1−	p53alt/WT1−	p53/WT1+	p53alt/WT1+
Type 2	96	10 (10.4%)	5 (5.2%)	20 (20.8%)	61 (63.5%)
Type 1	51	30 (58.8%)	13 (25.5%)	4 (7.8%)	4 (7.8%)
Borderline	49	12(24.5%)	11 (22.4%)	23 (46.9%)	3 (6.1%)
Comparison of stain patterns in type 2 and type 1 tumours					
Type 2 ovarian carcinomas			Type 1 ovarian carcinomas		
	

B. Altered p53 and WT1 expression differ significantly in OvCa according to FIGO stage (*n* = 196) (*p* < 0.0001)					
FIGO stage	*n*	p53/WT1−	p53alt/WT1−	p53/WT1+	p53alt/WT1+
FIGO IV	19	1 (5.3%)	3 (15.7%)	4 (21.1%)	11 (57.9%)
FIGO III	75	10 (13.3%)	5 (6.7%)	22 (29.3%)	38 (50.7%)
FIGO II	23	6 (26.1%)	6 (26.1%)	3 (13.0%)	8 (34.8%}
FIGO I	79	35(44.3%)	15 (19.0%)	18 (22.8%)	11 (13.9%)
Comparison of stain patterns in FIGO stages III and IV and FIGO stages I and II					
FIGO III and IV			FIGO I and II		
	

C. Altered p53 and WT1 expression differ significantly in OvCa according to tumour grade (*n* = 196) (*p* < 0.0001)					
Tumour grade	*n*	p53/WT1−	p53alt/WT1−	p53/WT1+	p53alt/WT1+
Grade 3	102	13 (12.8%)	7 (6.9%)	21 (20.6%)	61 (59.8%)
Grade 2	40	24 (60.0%)	11 (27.5%)	2 (5%)	3 (7.5%)
Grade 1	5	3 (60.0%)	0 (0%)	1 (20.0%)	1 (20.0%}
Borderline/LMP	49	12 (24.5%)	11 (22.4%)	23 (46.9%)	3 (6.2%)
Comparison of stain pattern in high grade vs. low grade ovarian carcinomas					
Grade 3 ovarian carcinomas			Grade 1 and 2 ovarian carcinomas		
	
D. Altered p53 and WT1 expression differ significantly in OvCa used in survival analyses according to subtype (*n* = 96) (*p* < 0.0001)					
Tumour subtype	*n*	p53/WT1−	p53alt/WT1−	p53/WT1+	p53alt/WT1+
Type 2	61	7 (11.5%)	2 (3.3%)	12 (19.7%)	40 (65.6%)
Type 1	35	21 (60%)	11 (31.4%)	3 (8.6%)	0 (0%)
Comparison of stain patterns in type 2 and type 1 tumours used in survival analyses					
Type 2 ovarian carcinomas			Type 1 ovarian carcinoma		
	

Data in Table 3A show that the percentages of type 1 and type 2 tumours with the four combinations of IHC detected p53 and WT1 nuclear expression differed significantly (*p* < 0.0001). These data suggested that the difference between type 2 and type 1 tumours is more highly related to expression of nuclear WT1 by type 2 tumours than to alterations in p53 nuclear stain. These data also suggested that, although as noted above, alterations in immunohistochemically detected p53 nuclear expression are associated with invasive tumours, these alterations in p53 expression are often associated with changes in WT1 expression in lethal type 2 tumours (see below and Fig. 2). These data give evidence for two pathways to lethal type 2 tumours; nuclear expression of WT1, and combined altered p53 and WT1 nuclear expression.

Data in Table 3B show that the percentages of carcinomas at late stages (FIGO III and IV) and at early stages (FIGO I and II) with the four combinations of expression of p53 and nuclear WT1 differed significantly (*p* < 0.0001). These data suggested that invasive ovarian cancer at early stages (FIGO I and II) are associated with p53 alterations, whereas the progression of ovarian cancer to distant metastases (FIGO III and IV) is associated with tumours having nuclear WT1 expression. These data also suggested that alterations in p53 transcription factor protein expression synergised with nuclear WT1 transcription protein expression in distant metastases clinically.

As seen in Table 3C, high-grade (grade 3) tumours and low-grade (grades 1 and 2) tumours differed significantly in the four combinations of expression of p53 and WT1 (*p* < 0.0001). These data (Table 3C) suggested that altered p53 expression is associated with WT1 in increased grade, dedifferentiated, ovarian tumours in women.

Taken together, these data showed that tumours that expressed normal p53 and were WT1 negative predominated in type 1, grades 1 and 2, and FIGO I and II OvCa. Tumours with altered p53 expression that were negative for WT1 were found mainly in type 1, grade 2 and FIGO II OvCa. Tumours with normal p53 that expressed WT1 were found mainly in type 2, grade 3 and FIGO III OvCa. Importantly, type 2, grade 3 and FIGO IV OvCa had both altered p53 and WT1 nuclear expression. Overall, OvCa that had both altered p53 transcription factor protein expression and WT1 transcription factor protein expression differed significantly from OvCa that had normal p53 expression and were negative for WT1 (*p* < 0.0001).

### Histopathologic subtypes, tumour grade, FIGO stage of disease and patient age modify survival from ovarian cancer

As suggested by 'REporting recommendations for tumour MARKer prognostic studies(REMARK)'^26^ we first studied the 137 ovarian tumours in our data set for standard prognostic variables including histopathologic subtype, tumour grade and stage of disease (Table 1B and Fig. 1b) for comparison to the proposed prognostic biomarkers of survival, namely altered immunohistochemically detected p53 and WT1 nuclear expression. Since only two patients with borderline tumours died of their disease, further survival analyses only considered the 96 patients with type 1 and type 2 OvCa.

Statistical analyses found that patient age was a confounding factor for survival analyses in the 137 tumours (Table 1B). Survival analyses on 96 patients with type 1 or type 2 OvCa (Fig. 1b) showed that OvCa type was significant (*p* = 0.0003). Median survival for patients with type 2 OvCa was 3.21 years while median survival for type 1 OvCa was at least 18.65 years. After adjusting for age, the hazard ratio for patients with type 2 vs. type 1 OvCa was 2.75 (*p* = 0.0011). Tumour grade was also significant (*p* = 0.0072). Median survival for patients with grade 3 OvCa was 3.35 years while median survival for those with grade 2 OvCa was at least 18.65 years. The hazard ratio for grade 3 vs. grade 2 OvCa was 2.54 (*p* = 0.0037) after adjusting for age. FIGO stage was significant (*p* < 0.0001). Median survival for patients at FIGO stages IV, III, II and I was 1.64, 2.37, 5.95 and at least 18.65 years, respectively. The hazard ratios for FIGO IV, III, II vs I were 6.32 (*p* < 0.0001), 6.62 (*p* < 0.0001), 2.45 (*p* = 0.0674) after adjusting for age. The hazard ratio for FIGO III, IV vs. II, I was 4.70 (*p* < 0.0001) after adjusting for age. Hazard ratio analysis demonstrated that tumour type and grade were highly associated, and that FIGO stage was more predictive than either tumour type or grade.

### p53 alterations are not significant for patient survival in a multivariant model

**Fig. 3:**
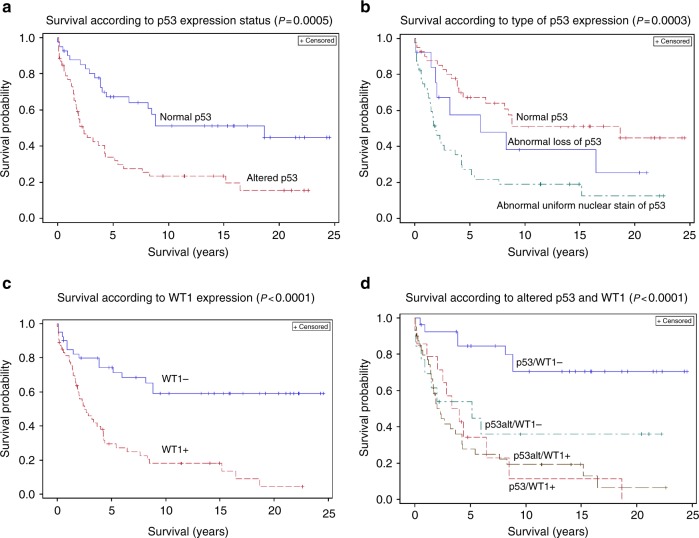
Clinical relevance of WT1 and altered p53 in OvCa. **a** Patient survival is highly dependent upon the altered expression of p53 proteins in invasive OvCa (type 1, indolent OvCa, and type 2, aggressive OvCa) (*p* = 0.0004). After adjusting for age and tumour type, the hazard ratio for altered p53 expression is 1.55. **b** The worse survival in patients with OvCa expressing abnormal uniform p53 nuclear stain vs. abnormal loss of nuclear p53 stain does not reach statistical significance (*p* = 0.1055). **c** Patient survival is highly dependent upon the expression of Wilms’ Tumour 1 (WT1) protein in OvCa (type 1 and type 2) (*p* = 0.0001). After adjusting for age and tumour type, the hazard ratio for WT1 expression is 2.25. **d** Co-expression of WT1 and altered p53 increases risk of death from invasive ovarian cancer (hazard ratio = 3.358 compared to cancers without either protein). Survival is significantly reduced when either WT1 or altered p53 are expressed (*p* = 0.0001); however, when WT1 is present, the adverse effect of altered p53 expression on survival is increased

Patients with invasive OvCa, type 1 or type 2, having any type of IHC detected altered p53 expression, irrespective of morphology, had a shorter survival than those with the wild-type, normal expression pattern (*p* = 0.0006) (Fig. 3a). Median survival was 2.30 and at least 18.65 years, respectively. However, after adjusting for age and tumour subtype (type 1 and type 2), the hazard ratio for altered p53 was 1.52 (*p* = 1.590) and therefore not significant. If we adjust for age and FIGO, the hazard ratio was 1.62, *p* = 0.0902. The apparent difference in survival between patients with type 1 and type 2 OvCa with the abnormal loss of p53 nuclear stain and the abnormal uniform nuclear p53 stain did not reach statistical significance (*p* = 0.1055) (Fig. 3b).

### WT1 expression is a poor prognostic factor for survival of patients with invasive ovarian carcinomas

Although all serous borderline tumours, which are non-invasive and are minimally deviated histologically from normal ovarian tissues, expressed WT1, these borderline tumours were not lethal. In marked contrast, WT1 expression in invasive ovarian carcinomas, irrespective of morphology, was highly associated with poor overall survival of patients (*p* < 0.0001) (Fig. 3c). Median survival was 2.30 and at least 8.84 years, respectively. After adjusting for age and tumour subtype, the hazard ratio for WT1 expression was 2.17 (*p* = 0.0589). After adjusting for age and FIGO, the hazard ratio was 1.82 (*p* = 0.0639). When WT1 and p53 were both in the age-adjusted Cox model, WT1 was significant (*p* = 0.0024), while p53 was not (*p* = 0.2144).

### Patterns of nuclear WT1 and p53 expression are related not only to ovarian tumour type, grade and FIGO stage, but also to survival

Table 3D gives the distribution of the markers in OvCa from the 96 patients used in the survival analysis. The data in Table 3D demonstrate that WT1 and altered p53 expression differed significantly in OvCa according to subtype in patients studied in the survival analyses.

Overall survival from ovarian cancer was highly related to altered p53 and WT1 nuclear expression (*p* < 0.0001, Fig. 3d). The median survival for patients with OvCa having abnormal p53 nuclear expression and that also expressed nuclear WT1 was 2.30 years. Median survival for patients with OvCa having normal p53 expression that also expressed WT1 was 4.00 years, and was 5.16 years for patients with OvCa having altered p53 nuclear expression but negative for WT. In contrast, median survival for patients with OvCa having normal p53 nuclear expression and without WT1 was 8.84 years. The 20-year survival probability of patients with OvCa without either an altered p53 nuclear stain pattern or nuclear WT1 expression was 67.7% and was significantly better than other patients (see Fig. 3d). The 20-year survival probability for patients with OvCa negative for WT1 but with altered p53 expression was 35.9%, and was 0% for patients with OvCa expressing WT1 and the normal p53 stain pattern. Patients with OvCa expressing both nuclear WT1 and altered p53 expression had a 20-year survival probability of 6.5%. If survival in patients with OvCa having either abnormal p53 nuclear stain pattern or nuclear WT1 expression were combined, then adjusted for age and tumour subtype the hazard ratio compared to patients with OvCa having normal p53 nuclear expression and negative for WT1 was 2.70 (*p* = 0.0201) and *p* = 0.0296 with a hazard ratio of 2.40 when age and FIGO adjusted. We conclude that altered p53 and nuclear WT1 expression combined was associated with reduced survival after adjusting for both age and tumour subtype as well as age and FIGO stage, and that nuclear WT1 expression was a biomarker for lethal OvCa and that OvCas expressing WT1 are more frequently lethal when p53 is also altered.

## Discussion

'Ovarian cancer' is a group of genetically, biochemically and morphologically different diseases. Not all invasive OvCas of any one morphological type are lethal. Patient survival depends upon the well-known prognostic factors of tumour type, tumour grade and FIGO stage. However, none of these prognostic factors offer a target for therapy. Here we report in a cohort of ovarian carcinoma patients with long-term follow-up that altered p53 and WT1 nuclear expression in invasive OvCa is associated with reduced survival after adjusting for both age and FIGO stage. Since both WT1 and p53 are tumour-associated antigens and WT1 is ranked first in pilot prioritisation out of 75 cancer antigens based on predefined criteria including therapeutic efficacy and immunogenicity,^13^ these proteins offer targets for therapy of lethal OvCa. However, in this cohort of OvCa patients with long-term follow-up, abnormal p53 expression detected immunohistochemically does not predict survival based on multivariate analysis.

The WT1 is a transcription factor protein involved in the transcriptional regulation of genes such as growth factors, regulators of the cell cycle as well as apoptosis, and differentiation markers.^27^ WT1 expression is increased during progression of OvCa to metastasis.^27,28^ Silencing of WT1 in OvCa cell lines with siRNA reduces OvCa cell motility and ability to invade 3D collagen-rich matrices.^28^ Conversely, forced overexpression of WT1 in an OvCa cell line increased cell invasion in a Boyden chamber assay as well as cell proliferation.^27^

WT1 and p53 are transcription factors and tumour-associated antigens that are differentially co-expressed in all subtypes of OvCa. In multiple experimental models, p53 and WT1 are known to interact physically and functionally. Maheswaran et al.^19^ proposed that under conditions of partial or total p53 tumour functional inactivation, the WT1 protein could be converted from a transcriptional repressor to an activator. Viel et al.^29^ also suggested that when wild-type p53 is scarce or absent, an overproduced WT1 protein could acquire activational properties, thus completely reversing its transcriptional control of growth-related genes. Consistent with these studies, altered p53 expression combined with WT1 expression predominated in type 2, grade 3 and FIGO IV ovarian carcinomas. Moreover, we find that altered p53 expression and WT1 expression combined in OvCa is a better predictor of patient survival than tumour type or tumour grade. The effect of ovarian cancer expression of altered p53 and/or WT1 is associated with an increased risk of death after adjusting for both age and FIGO stage when compared to patients with tumours with normal p53 expression and a lack of nuclear WT1 expression (hazard ratio = 2.4).

In contrast to tumour type, grade and FIGO stage, immunohistochemically detected WT1 and altered p53 expression are tumour-associated antigens and transcription factors that, as shown here, identify tumours with an aggressive phenotype and also provide a potential target for immunotherapy. Identifying tumours with a more aggressive phenotype will lead to a rational selection of patients for immunotherapy.

The hazard ratio for death in patients with tumours having WT1 expression but without altered p53 or with both WT1 and altered p53 expression was 3.252 and 3.358, respectively, compared to patients with tumours without either WT1 or altered p53 expression. Vermeij et al.^30^ demonstrated that WT1 overexpression in ovarian tumours was associated with intratumoural Treg and cytotoxic T cell infiltration, which was an independent prognostic factor for progression-free survival.

Clinical trials of WT1 immunotherapy suggested that WT1 is a strong tumour-associated antigen that can by itself induce some immunological and oncological changes.^31,32^ However, additional approaches such as combined vaccination with other tumour-associated antigens, or standard chemotherapy, or antibodies against immune checkpoints will be needed to overcome the immunosuppressive factors in the ovarian cancer microenvironment.^32–34^ The hope is that a combination of Treg targeting, along with the activation of tumour-specific effector T cells by cancer vaccination or immune checkpoint blockade, will make cancer immunotherapy more efficacious.^34^ As recently reviewed,^35^ response to immune checkpoint blockade depends not only on the molecular characteristics of the tumour, but also on the tumour microenvironment, the immune competence of the patient and environmental influences such as the patient’s gut microbiome.

Our data are consistent with the conclusions that: (1) invasive ovarian cancers at early stages (FIGO I and II) are associated with altered nuclear p53 expression, whereas metastatic ovarian cancers (FIGO III and IV) are associated with nuclear WT1 expression; (2) compared to patients without either WT1 or altered p53 expression, patients with tumours expressing altered p53 or nuclear WT1 have decreased survival even when these data are adjusted for age and tumour type or age and FIGO stage; and (3) the transcription factor(s) WT1, or WT1 and altered p53 expression are prognostic biomarkers of poor survival and could be useful predictive biomarkers for stratifying patients eligible for new immunologic approaches targeting the tumour-associated antigen WT1 for therapy of lethal ovarian cancers.

## Availability of data

The data generated during this study are available from the corresponding author on reasonable request.
